# Supplementary material for: A predictive approach to enhance time-series forecasting
Source: Nat Commun. 2025 Sep 30;16:8645. doi: 10.1038/s41467-025-63786-4 (PMC12485041; doi:10.1038/s41467-025-63786-4)
Supplement: Supplementary file 1 — Supplementary Infomation [file 41467_2025_63786_MOESM1_ESM.pdf]

Supplemental Information: A Predictive Approach To Enhance Time-Series Forecasting

Skye Gunasekaran[1], Assel Kembay[1], Hugo Ladret[2], Rui-Jie Zhu[1], Laurent Perrinet[3], Omid Kavehei[4], Jason Eshraghian[1*]

[1]Department of Electrical and Computer Engineering, University of California, Santa Cruz, CA, USA
[2]Friedrich Miescher Institute for Biomedical Research, Basel, Switzerland
[3]Institut de Neurosciences de la Timone, Aix Marseille Univ, CNRS, Marseille, France
[4]School of Biomedical Engineering, The University of Sydney, Sydney, Australia
[*] Corresponding author: jsn@ucsc.edu

Additional Seizure Information

Epilepsy Background Information

Epilepsy is a neurological condition which affects millions of people worldwide. It is characterized by a rapid firing in brain activity, often leading to spasms and involuntary muscle movements. Events of epilepsy are referred to as "ictal" events, whereas pre-seizure and non-seizure periods are referred to as "preictal" and "interictal", respectively [1].

Due to the unpredictable nature of seizures, there has been a widespread effort for decades to create accurate seizure prediction mechanisms [2, 3]. However, attempts to do so have faced many challenges due to patient specificity, high noise of signals, and sparseness of seizure events. To further complicate the task, there has been contradicting evidence regarding the relationship between preictal and ictal events [4].

Patients with epilepsy who are undergoing medical treatment are typically monitored via scalp EEG, in which a varying number of nodes are placed on the patients head according to the 10-20 international standard [5]. Clinicians are tasked with

monitoring these signals actively, and can sound an alarm when a seizure takes place to alert medical staff. Therefore, having an accurate seizure forecasting mechanism can help alleviate this burden on hospitals [4].

Seizure Data Preprocessing

Our preprocessing pipeline of EEG data is as follows. Patient data is classified into three separate categories: interictal, preictal, and ictal. With respect to preictal data, we use a seizure occurrence period of 30 minutes, and a seizure prediction horizon of 5 minutes. Data up to 5 minutes prior to seizure onset is concealed from the student, and the 30 minutes prior to this is labeled as preictal data [6]. Due to this restriction, patients whose preictal data was not long enough were omitted from the dataset.

Each data sample undergoes a short-time-fourier transform (STFT) with a window size of 30 seconds. On both preictal and ictal data, we apply oversampling by taking a sliding window of STFTs. These overlapping samples are removed in the testing set. Our train-test-split was created by concatenating all of the interictal data, and evenly distributing them between ictal or preictal periods. The final 35% of seizures, as well as their corresponding interictal data, are used for testing. No shuffling is performed to preserve the temporal order of data.

Experimental Details

Dataset splits. For each patient we gather *all* annotated EEG segments:

1. every inter-ictal (I) segment, and
2. every ictal *or* pre-ictal (S) segment.

We then build a **balanced, chronological stream**

$$\mathrm{I}_1\ \mathrm{S}_1\ \mathrm{I}_2\ \mathrm{S}_2\ \ldots$$

by interleaving the two classes so that each seizure block S_j is immediately preceded by the amount of inter-ictal data required to keep the cumulative class counts equal.[1] This balanced stream is split *contiguously*: the first 65 % forms the training set and the final 35 % the held-out test set. No window, channel, or seizure appears in both splits.

Models compared:

- **Baseline (CNN–LSTM).** batch-norm $\rightarrow$ Conv3D($1 \rightarrow 16,\ k = (T/2, 5, 5), s = (1, 2, 2)$) $\rightarrow$ max-pool $\rightarrow$ batch-norm $\rightarrow$ Conv3D($16 \rightarrow 32, k = 3, s = 1$) $\rightarrow$ avg-pool$(3, 2)$ $\rightarrow$ 3-layer LSTM (512 hidden) $\rightarrow$ FC 512 to FC 256 to 2.
- **FGL (ours).** Teacher: Seizure-detector (CNN-LSTM Backbone) Student: Seizure-predictor (CNN-LSTM Backbone) Trained with the Future-Guided loss (Eq.1); $\alpha \in \{0, 0.1, \ldots, 1\}$.
- **MViT.** Multi-scale Vision Transformer per EEG channel: patch size $(5, 10)$, embed dim 128, 4 heads, hidden dim 256, 4 encoder layers, dropout 0.1. Channel embeddings are concatenated and fed to a linear head ($128 \times C \rightarrow 2$).

[1] The first block is always inter-ictal because patients typically enter the hospital in a non-seizure state.

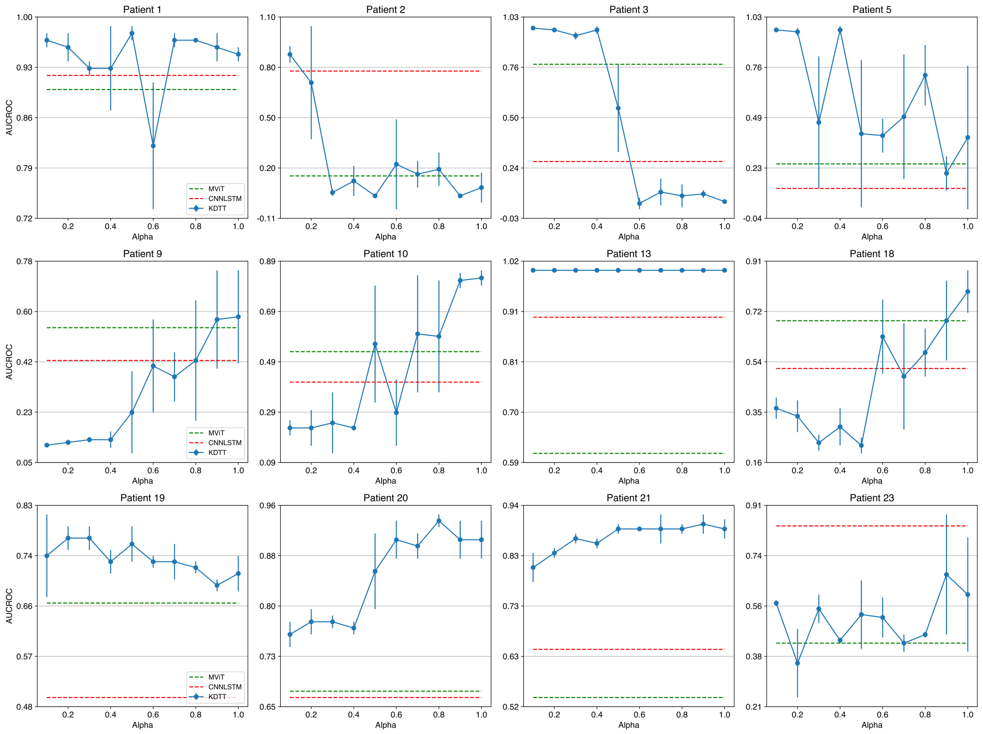

Supplementary Figure 1: **CHBMIT ablation study on alpha.** MViT results are represented as a dashed red line, and CNNLSTM results as a dashed green line.

Optimisation and over-fitting control: All models are trained with Adam (lr $= 5\times10^{-4}$, $\beta_1 = 0.9$, $\beta_2 = 0.999$, $\varepsilon = 10^{-8}$); batch size 32; maximum 25 epochs. To guard against overfitting, each experiment is repeated with multiple random initializations on the same dataset, and stability is assessed across runs.

Evaluation: For each patient we conduct three independent trials and report mean $\pm$ std. of sensitivity, false-positive rate (FPR), and AUC–ROC on the test split. The decision threshold is chosen per patient via Youden's J statistic. Results appear in Table 1; best numbers per patient are shown in **bold**. **Hardware:** All models were trained using an NVIDIA RTX 4080 GPU.

Mackey Glass Experiments

Experimental Details

The Mackey-Glass (MG) equation is a delay differential equation used to model chaotic time-series data. It is defined as follows:

$$\frac{dP(t)}{dt} = \frac{\beta_0 \theta^n P(t-\tau)}{\theta^n + P(t-\tau)^n} - \gamma P(t) \tag{1}$$

where:

Supplementary Table 1: CHBMIT Comparison with SOTA

	MViT			CNNLSTM			FGL		
Patient	FPR	Sensitivity	AUCROC	FPR	Sensitivity	AUCROC	FPR	Sensitivity	AUCROC
1	$\mathbf{0.02}_{\pm 0.02}$	$0.82_{\pm 0.22}$	$0.90_{\pm 0.12}$	$0.11_{\pm 0.12}$	$0.88_{\pm 0.10}$	$0.92_{\pm 0.07}$	$0.04_{\pm 0.01}$	$\mathbf{0.89}_{\pm 0.04}$	$\mathbf{0.98}_{\pm 0.01}$
2	$0.63_{\pm 0.45}$	$0.66_{\pm 0.47}$	$0.15_{\pm 0.19}$	$0.35_{\pm 0.33}$	$\mathbf{0.89}_{\pm 0.15}$	$0.78_{\pm 0.17}$	$\mathbf{0.10}_{\pm 0.04}$	$0.83_{\pm 0.18}$	$\mathbf{0.88}_{\pm 0.05}$
3	$0.16_{\pm 0.04}$	$0.66_{\pm 0.23}$	$0.78_{\pm 0.15}$	$0.42_{\pm 0.40}$	$0.49_{\pm 0.41}$	$0.27_{\pm 0.19}$	$\mathbf{0.01}_{\pm 0.01}$	$\mathbf{0.90}_{\pm 0.02}$	$\mathbf{0.97}_{\pm 0.01}$
5	$0.61_{\pm 0.43}$	$0.66_{\pm 0.47}$	$0.25_{\pm 0.10}$	$0.65_{\pm 0.46}$	$0.67_{\pm 0.47}$	$0.12_{\pm 0.10}$	$\mathbf{0.07}_{\pm 0.00}$	$\mathbf{0.97}_{\pm 0.01}$	$\mathbf{0.96}_{\pm 0.01}$
9	$\mathbf{0.20}_{\pm 0.16}$	$0.41_{\pm 0.41}$	$0.54_{\pm 0.21}$	$0.64_{\pm 0.45}$	$0.67_{\pm 0.47}$	$0.42_{\pm 0.10}$	$0.60_{\pm 0.04}$	$\mathbf{0.94}_{\pm 0.02}$	$\mathbf{0.57}_{\pm 0.18}$
10	$\mathbf{0.25}_{\pm 0.17}$	$0.48_{\pm 0.13}$	$0.53_{\pm 0.17}$	$0.56_{\pm 0.38}$	$0.61_{\pm 0.34}$	$0.41_{\pm 0.13}$	$0.26_{\pm 0.04}$	$\mathbf{0.65}_{\pm 0.07}$	$\mathbf{0.81}_{\pm 0.03}$
13	$0.18_{\pm 0.14}$	$0.67_{\pm 0.36}$	$0.61_{\pm 0.32}$	$0.11_{\pm 0.10}$	$\mathbf{0.99}_{\pm 0.01}$	$0.90_{\pm 0.10}$	$\mathbf{0.00}_{\pm 0.00}$	$\mathbf{0.99}_{\pm 0.01}$	$\mathbf{1.00}_{\pm 0.00}$
18	$\mathbf{0.30}_{\pm 0.10}$	$0.68_{\pm 0.15}$	$\mathbf{0.69}_{\pm 0.07}$	$0.54_{\pm 0.28}$	$0.64_{\pm 0.26}$	$0.51_{\pm 0.03}$	$0.43_{\pm 0.14}$	$\mathbf{0.88}_{\pm 0.06}$	$\mathbf{0.69}_{\pm 0.15}$
19	$\mathbf{0.07}_{\pm 0.05}$	$0.47_{\pm 0.33}$	$0.66_{\pm 0.23}$	$0.80_{\pm 0.04}$	$\mathbf{0.97}_{\pm 0.01}$	$0.50_{\pm 0.07}$	$0.48_{\pm 0.10}$	$0.89_{\pm 0.07}$	$\mathbf{0.76}_{\pm 0.03}$
20	$0.38_{\pm 0.07}$	$\mathbf{0.98}_{\pm 0.03}$	$0.67_{\pm 0.09}$	$0.45_{\pm 0.03}$	$0.95_{\pm 0.06}$	$0.66_{\pm 0.07}$	$\mathbf{0.13}_{\pm 0.03}$	$0.86_{\pm 0.02}$	$\mathbf{0.94}_{\pm 0.01}$
21	$0.60_{\pm 0.03}$	$\mathbf{1.00}_{\pm 0.00}$	$0.54_{\pm 0.03}$	$0.49_{\pm 0.14}$	$\mathbf{0.97}_{\pm 0.02}$	$0.64_{\pm 0.18}$	$\mathbf{0.12}_{\pm 0.02}$	$0.73_{\pm 0.04}$	$\mathbf{0.90}_{\pm 0.02}$
23	$0.62_{\pm 0.06}$	$\mathbf{1.00}_{\pm 0.00}$	$0.43_{\pm 0.02}$	$\mathbf{0.20}_{\pm 0.27}$	$0.92_{\pm 0.12}$	$\mathbf{0.84}_{\pm 0.22}$	$0.50_{\pm 0.08}$	$\mathbf{1.00}_{\pm 0.00}$	$0.67_{\pm 0.21}$
AVG	$0.33_{\pm 0.21}$	$0.70_{\pm 0.19}$	$0.56_{\pm 0.20}$	$0.44_{\pm 0.20}$	$0.80_{\pm 0.16}$	$0.58_{\pm 0.24}$	$\mathbf{0.22}_{\pm 0.01}$	$\mathbf{0.87}_{\pm 0.02}$	$\mathbf{0.84}_{\pm 0.15}$

Supplementary Table 2: AES Comparison with SOTA

	MViT			CNNLSTM			FGL		
Patient	FPR	Sensitivity	AUCROC	FPR	Sensitivity	AUCROC	FPR	Sensitivity	AUCROC
Dog 1	$0.63_{\pm 0.06}$	$0.93_{\pm 0.05}$	$0.57_{\pm 0.00}$	$0.62_{\pm 0.02}$	$\mathbf{1.00}_{\pm 0.00}$	$0.61_{\pm 0.01}$	$\mathbf{0.37}_{\pm 0.05}$	$0.99_{\pm 0.01}$	$\mathbf{0.81}_{\pm 0.01}$
Dog 2	$\mathbf{0.22}_{\pm 0.07}$	$0.48_{\pm 0.04}$	$0.62_{\pm 0.01}$	$0.34_{\pm 0.03}$	$\mathbf{0.93}_{\pm 0.03}$	$\mathbf{0.83}_{\pm 0.00}$	$0.40_{\pm 0.02}$	$\mathbf{0.93}_{\pm 0.01}$	$\mathbf{0.83}_{\pm 0.00}$
Dog 3	$0.82_{\pm 0.16}$	$\mathbf{0.87}_{\pm 0.17}$	$0.49_{\pm 0.02}$	$0.35_{\pm 0.17}$	$0.73_{\pm 0.05}$	$0.75_{\pm 0.07}$	$\mathbf{0.20}_{\pm 0.08}$	$0.71_{\pm 0.07}$	$\mathbf{0.87}_{\pm 0.01}$
Dog 4	$0.36_{\pm 0.08}$	$0.66_{\pm 0.08}$	$0.70_{\pm 0.02}$	$\mathbf{0.35}_{\pm 0.05}$	$0.83_{\pm 0.05}$	$0.77_{\pm 0.01}$	$\mathbf{0.35}_{\pm 0.02}$	$\mathbf{0.87}_{\pm 0.02}$	$\mathbf{0.79}_{\pm 0.01}$
Dog 5	$0.37_{\pm 0.04}$	$0.93_{\pm 0.02}$	$0.75_{\pm 0.02}$	$0.47_{\pm 0.03}$	$\mathbf{0.97}_{\pm 0.01}$	$0.70_{\pm 0.01}$	$\mathbf{0.27}_{\pm 0.07}$	$0.88_{\pm 0.00}$	$\mathbf{0.85}_{\pm 0.02}$
Human 1	$\mathbf{0.20}_{\pm 0.07}$	$0.89_{\pm 0.03}$	$\mathbf{0.91}_{\pm 0.04}$	$0.51_{\pm 0.16}$	$0.91_{\pm 0.09}$	$0.65_{\pm 0.17}$	$0.28_{\pm 0.01}$	$\mathbf{0.96}_{\pm 0.02}$	$0.85_{\pm 0.00}$
Human 2	$0.98_{\pm 0.00}$	$\mathbf{1.00}_{\pm 0.00}$	$0.35_{\pm 0.01}$	$\mathbf{0.21}_{\pm 0.08}$	$0.82_{\pm 0.10}$	$0.86_{\pm 0.07}$	$0.28_{\pm 0.13}$	$0.90_{\pm 0.06}$	$\mathbf{0.93}_{\pm 0.02}$
AVG	$0.51_{\pm 0.28}$	$0.82_{\pm 0.16}$	$0.63_{\pm 0.15}$	$0.40_{\pm 0.12}$	$0.88_{\pm 0.08}$	$0.78_{\pm 0.06}$	$\mathbf{0.30}_{\pm 0.02}$	$\mathbf{0.89}_{\pm 0.01}$	$\mathbf{0.85}_{\pm 0.01}$

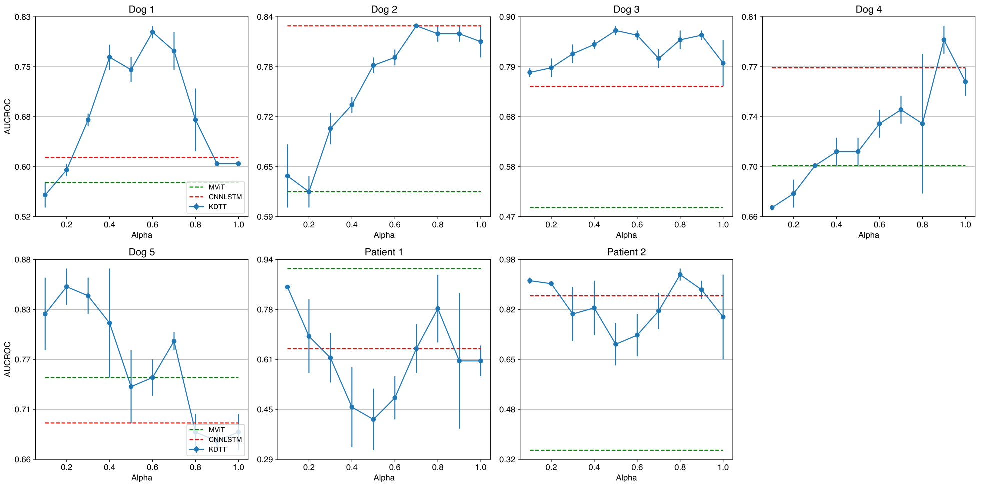

Supplementary Figure 2: **AES ablation study on alpha.** MViT results are represented as a dashed red line, and CNNLSTM results as a dashed green line.

- $P(t)$: The state variable at time t.
- τ: The time delay parameter.
- β_0: The growth rate parameter.
- θ: The scaling parameter.
- n: The exponent controlling the nonlinearity.
- γ: The decay rate.

For our experiments, we used the following parameter values:

- $\tau = 17$
- Initial condition $P(0) = 0.9$
- $n = 10$
- $\beta_0 = 0.2$
- $\gamma = 0.1$
- Time step size $dt = 1.0$
- Lookback window $= 8$

Dataset splits. Given the parameters above, we generate a trajectory of length 10 000, use the first 6 000 points for training, the next 2 000 points for validation, and the final 2 000 for testing (60% / 20% / 20%).

Models compared:

- **Baseline (RNN).** RNN($8 \to bin\_size, h = 128, l = 2$) $\to$ FC 128 to FC 128 to bin_size.
- **FGL (ours).** Teacher: 1-step predictor (RNN Backbone) Student: N-step predictor (RNN Backbone) Trained with the Future-Guided loss (Eq. 1); $\alpha \in \{0 \dots 1\}$.

Optimization and over-fitting control: All models are trained with Adam (lr $= 1 \times 10^{-4}$, $\beta_1 = 0.9$, $\beta_2 = 0.999$; batch size 128). We employ dropout (rate $= 0.2$)

for regularization, fix the training budget to 50 epochs, and initialize random seeds at the start of each training regime. Early stopping (patience = 5 epochs, tolerance = 1×10^{-4}) is applied by monitoring performance on the validation set each epoch, and the best checkpoint is restored before final evaluation. **Hardware:** All models were trained using an NVIDIA RTX 4080 GPU.

Mackey Glass Dataset Results

Supplementary Table 3: FGL Results on Mackey–Glass

	**Bins = 25**			**Bins = 50**		
**Horizon**	**Baseline**	$\alpha = 0.5$	$\alpha = 0.0$	**Baseline**	$\alpha = 0.5$	$\alpha = 0.0$
2	1.55	1.44	1.44	5.07	4.55	5.20
3	2.65	2.27	2.27	9.38	8.05	7.96
4	3.60	3.12	3.16	12.61	10.67	10.77
5	4.94	4.29	4.30	17.64	14.42	13.86
6	6.34	5.37	6.86	20.61	17.51	17.42
7	5.83	7.45	5.60	23.61	18.37	19.01
8	6.37	5.80	5.63	30.71	17.34	18.59
9	6.18	5.14	5.00	20.79	16.49	14.45
10	5.21	3.70	3.98	16.94	11.56	12.75
11	3.78	2.83	3.18	14.60	9.00	9.73
12	3.04	2.39	2.66	11.48	8.67	9.58
13	2.82	2.14	2.36	195.62	9.39	9.90
14	2.61	2.02	2.30	9.53	9.06	8.73
15	2.30	1.91	2.25	8.22	8.44	9.35
**Avg**	4.09	**3.56**	3.64	28.34	**11.68**	11.95

Page Hinkley Drift Adaptation Experiment

In this supplementary experiment, we evaluate whether post-hoc drift adaptation-specifically a Page–Hinkley-can compensate for FGL. To do so, we take our three Mackey–Glass forecasting models (the baseline, the FGL student with $\alpha = 0.0$, and the FGL student with $\alpha = 0.5$), and expose each to identical PH retraining. By showing that the relative performance ranking established by FGL during training persists even after this adaptive correction, we demonstrate that the advantages of future-guided learning are not supplanted by simple post-hoc methods, but rather remain an independent—and complementary—means of improving forecast accuracy.

Let e_t be the instantaneous forecast error (MSE) at time t, and let

$$\bar{e}_t = \frac{1}{t} \sum_{i=1}^{t} e_i$$

be its cumulative running mean. The PH test tracks the one-sided cumulative deviation

$$u_t = e_t - \bar{e}_{t-1} - \delta, \quad S_t = \sum_{i=1}^{t} u_i, \quad S_{\min}(t) = \min_{1 \le i \le t} S_i.$$

A drift alarm is raised whenever

$$S_t - S_{\min}(t) > \lambda,$$

where $\delta > 0$ is a small tolerance (to avoid false alarms) and $\lambda > 0$ is the detection threshold. In our setup we use a sliding window of length 3 to recompute $\bar{e}$ and reset S_t after each retraining, and we choose (δ, λ) per bin-count. Upon alarm, we retrain each model for 3 epochs on the most recent data to correct for drift.

Supplementary Table 4: FGL Results on Mackey–Glass w/ Page Hinkley

	Bins = 25			Bins = 50		
**Horizon**	**Baseline**	$\alpha = 0.5$	$\alpha = 0.0$	**Baseline**	$\alpha = 0.5$	$\alpha = 0.0$
2	1.59	1.40	1.41	5.65	4.45	4.96
3	2.58	2.22	2.25	9.43	7.12	7.18
4	3.55	3.23	3.23	12.52	10.59	10.74
5	4.90	4.35	4.23	18.22	14.09	14.38
6	6.38	5.12	5.09	20.86	17.12	17.59
7	6.13	7.58	5.65	23.13	18.35	18.84
8	6.65	5.47	5.98	31.78	17.75	17.36
9	5.72	4.90	4.80	20.71	14.79	13.52
10	4.79	3.50	3.59	17.94	11.72	12.28
11	3.61	2.70	2.86	13.58	9.57	10.35
12	3.22	2.29	2.44	11.01	8.73	10.02
13	2.77	2.06	2.28	223.29	8.71	9.63
14	2.41	1.91	2.15	203.58	8.18	9.27
15	2.22	1.84	2.07	195.71	7.26	9.66
**Avg**	4.04	3.47	**3.43**	57.67	**11.32**	11.84

Table 1 demonstrates that, even after excluding non-convergent horizons, FGL with $\alpha = 0.5$ and $\alpha = 0.0$ both deliver substantial reductions in average MSE relative to the baseline. For Bins = 25, FGL reduces MSE by 14.1% ($\alpha = 0.5$) & 15.1% ($\alpha = 0.0$) when PH is enabled, and by 13.0% and 11.0% without PH. For Bins = 50, the corresponding improvements are 27.3% & 25.8% (with PH) and 23.4% & 21.8% (without PH). These results confirm that FGL's advantage holds consistently across both α settings and that applying PH on top of FGL yields an additional 2–3% MSE reduction, underscoring their complementary nature.

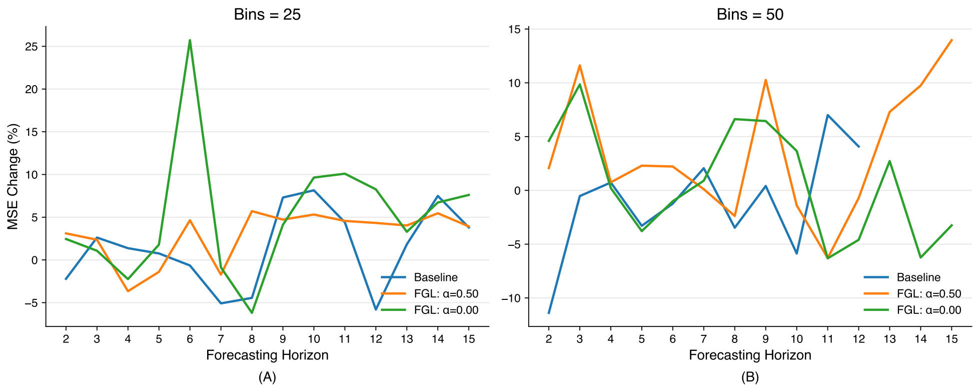

Supplementary Figure 3: **Application of Page–Hinkley (PH) drift adaptation to FGL.** We applied the same online PH detector (3-sample sliding window, 3-epoch retraining) to three Mackey–Glass forecasters: Baseline (no FGL), FGL ($\alpha = 0$), and FGL ($\alpha = 0.5$). PH sensitivity parameters were tuned per bin count (25 bins: $\delta = 0.130$, $\lambda = 0.647$; 50 bins: $\delta = 5.78$, $\lambda = 7.84$), while all other hyperparameters match the original experiments. Each panel shows the per-horizon percentage change in MSE after retraining relative to the original forecasts. Both FGL students ($\alpha = 0.0$ and $\alpha = 0.5$) show a meaningful average reduction in MSE loss, whereas the baseline's error actually increases. This demonstrates that training-time future guidance provides benefits that complement—not replace—post-hoc drift adaptation.

Future Guided Learning and Predictive Coding

To connect FGL to predictive coding, we review the mathematical intuition behind this theory, as explored in prior work [7, 8]. Using the common mathematical formalisms from these publications, we summarize the relevant concepts in this Appendix to establish a link between predictive processing and FGL. Predictive coding networks can be defined as ensembles of units that compute the posterior probabilities of environmental states $p(v|u)$ based on a top-down prior $p(v)$ and a bottom-up likelihood $p(u|v)$. Assuming Gaussian distributions for these terms, Bayes' theorem allows this computation to be expressed as:

$$p(v|u) = \frac{p(v)p(u|v)}{p(u)} = \frac{\frac{1}{\sqrt{2\pi\Sigma_p}} \exp\left(-\frac{(v-v_p)^2}{2\Sigma_p}\right) \frac{1}{\sqrt{2\pi\Sigma_u}} \exp\left(-\frac{(u-g(v))^2}{2\Sigma_u}\right)}{\int p(v)p(u|v), dv}. \quad (2)$$

Here, v represents a scalar value of the environment, characterized by v_p and Σ_p, the mean and variance of its Gaussian distribution, respectively. Similarly, $p(u|v)$ represents the likelihood, where the scalar u is modeled as the output of an activation function $g(v)$ (e.g., the reflection of light from a surface) with variance Σ_u.

The denominator in Equation 2 serves as a normalization term that is computationally intractable for biological neural networks due to the need to integrate over

all possible combinations of $p(v)$ and $p(u|v)$. One possible simplification is maximum likelihood estimation (MLE), which identifies the value of v that maximizes $p(v|u)$. This value, denoted as Φ, effectively replaces $p(v)$ in an MLE framework. Since the denominator is absent from the left-hand side of Equation 2 under MLE, we can instead focus on the numerator and express its logarithm, F, as:

$$F = \ln\left(p(\Phi)p(u|\Phi)\right) = \ln p(\Phi) + \ln p(u|\Phi). \tag{3}$$

We thus derive:

$$\begin{aligned}
F &= \ln p(\Phi) + \ln p(u|\Phi) \\
&= \ln\left(\frac{1}{\sqrt{2\pi\Sigma_p}}\exp -\frac{(\Phi - v_p)^2}{2\Sigma_p}\right) + \ln\left(\frac{1}{\sqrt{2\pi\Sigma_u}}\exp -\frac{(u - g(\Phi))^2}{2\Sigma_u}\right) \\
&= \ln\left(\frac{1}{\sqrt{2\pi\Sigma_p}}\right) + \ln\left(\exp -\frac{(\Phi - v_p)^2}{2\Sigma_p}\right) + \ln\left(\frac{1}{\sqrt{2\pi\Sigma_u}}\right) + \ln\left(\exp -\frac{(u - g(\Phi))^2}{2\Sigma_u}\right) \\
&= \ln\left(\frac{1}{\sqrt{2\pi}}\right) - \frac{1}{2}\ln\Sigma_p - \frac{(\Phi - v_p)^2}{2\Sigma_p} + \ln\left(\frac{1}{\sqrt{2\pi}}\right) - \frac{1}{2}\ln\Sigma_u - \frac{(u - g(\Phi))^2}{2\Sigma_u} \\
&= \frac{1}{2}\left(-\ln\Sigma_p - \frac{(\Phi - v_p)^2}{\Sigma_p} - \ln\Sigma_u - \frac{(u - g(\Phi))^2}{\Sigma_u}\right) + C
\end{aligned} \tag{4}$$

Where C is a constant combining terms that do not depend on Φ.
To determine the maximum likelihood estimate, we compute the derivative of F with respect to the surrogate value Φ:

$$\begin{aligned}
\frac{\delta F}{\delta \Phi} &= \frac{1}{2}\left(\frac{\delta}{\delta\Phi}\left(-\frac{(u - g(\Phi))^2}{\Sigma_u}\right) + \frac{\delta}{\delta\Phi}\left(-\frac{(\Phi - v_p)^2}{\Sigma_p}\right) + \frac{\delta}{\delta\Phi}\left(-\ln\Sigma_u\right) + \frac{\delta}{\delta\Phi}\left(-\ln\Sigma_p\right) + \frac{\delta}{\delta\Phi}C\right) \\
&= \frac{1}{2}\left(\left(-\frac{1}{\Sigma_u}\frac{\delta}{\delta\Phi}(u - g(\Phi))^2\right) + \left(-\frac{1}{\Sigma_p}\frac{\delta}{\delta\Phi}(\Phi - v_p)^2\right)\right)
\end{aligned} \tag{5}$$

Applying the power rule $(f(x)^n)' = nf(x)^{n-1}f'(x)$:

$$\begin{aligned}
\frac{\delta F}{\delta \Phi} &= \frac{1}{2}\left(\left(-\frac{1}{\Sigma_u}2(u-g(\Phi))\frac{\delta}{\delta\Phi}(u-g(\Phi))\right)+\left(-\frac{1}{\Sigma_p}2(\Phi-v_p)\frac{\delta}{\delta\Phi}(\Phi-v_p)\right)\right)\\
&= \frac{1}{2}\left(\left(-\frac{1}{\Sigma_u}2(u-g(\Phi))\left(\frac{\delta}{\delta\Phi}u-\frac{\delta}{\delta\Phi}g(\Phi)\right)\right)+\left(-\frac{1}{\Sigma_p}2(\Phi-v_p)\left(\frac{\delta}{\delta\Phi}\Phi-\frac{\delta}{\delta\Phi}v_p\right)\right)\right)\\
&= \frac{1}{2}\left(\left(-\frac{1}{\Sigma_u}2(u-g(\Phi))(-g'(\Phi))\right)+\left(-\frac{1}{\Sigma_p}2(\Phi-v_p)\right)\right)\\
&= \left(\frac{1}{\Sigma_u}(u-g(\Phi))(g'(\Phi))\right)+\left(-\frac{1}{\Sigma_p}(\Phi-v_p)\right)\\
&= \frac{(u-g(\Phi))}{\Sigma_u}g'(\Phi)+\frac{(v_p-\Phi)}{\Sigma_p}
\end{aligned} \tag{6}$$

For simplicity, we rewrite the terms as:

$$\begin{aligned}
\epsilon_p &= \frac{(v_p-\Phi)}{\Sigma_p}\\
\epsilon_u &= \frac{(u-g(\Phi))}{\Sigma_u},
\end{aligned} \tag{7}$$

where ϵ_p represents the prediction error on the causes, and ϵ_u represents the prediction error on the states [9]. Using these terms, the derivative of F with respect to Φ becomes:

$$\frac{\delta F}{\delta \Phi} = \epsilon_u g'(\Phi) + \epsilon_p \tag{8}$$

This gradient can be reformulated as a learning rule:

$$\dot{\Phi} = \epsilon_u g'(\Phi) - \epsilon_p \tag{9}$$

Conceptually, ϵ_p quantifies the prediction error on the causes, capturing the difference between the inferred value (Φ) and the model's prior expectation (v_p) [7]. In contrast, ϵ_u measures the prediction error on the states, reflecting the discrepancy between the observed value (u) and the predicted value ($g(\Phi)$). Simply put, ϵ_p corresponds to errors at higher-level representations, while ϵ_u pertains to raw differences between inferred and actual sensory inputs.

The function F, referred to as the variational free energy, serves as a key metric in predictive coding frameworks. While a detailed discussion is beyond the scope of this appendix, F intuitively relates to information-theoretic concepts, particularly as a lower bound on the model's surprise or uncertainty [9].

In predictive coding, one of the goals of a model is to maximize prediction efficiency and thus minimize both sources of prediction errors. At their minimal values, these errors converge to stable points, ϵ_p and ϵ_u, defined as:

$$\begin{aligned} \epsilon_p &= \frac{\Phi - v_p}{\Sigma_p} \\ \Sigma_p \epsilon_p &= \Phi - v_p \\ \Phi - v_p - \Sigma_p \epsilon_p &= 0 \end{aligned} \tag{10}$$

$$\begin{aligned} \epsilon_u &= \frac{u - g(\Phi)}{\Sigma_u} \\ \Sigma_u \epsilon_u &= u - g(\Phi) \\ u - g(\Phi) - \varSigma_u \varepsilon_u &= 0 \end{aligned} \tag{11}$$

The dynamics of a system aiming to minimize prediction errors and infer the most likely value of Φ under a MLE approach can then be described as:

$$\begin{aligned} \dot{\varepsilon_p} &= \Phi - v_p - \varSigma_p \varepsilon_p \\ \dot{\varepsilon_u} &= u - g(\Phi) - \varSigma_u \varepsilon_u \end{aligned} \tag{12}$$

This establishes an intuitive relationship between predictive coding and FGL. In predictive coding, the uncertainties $\varSigma_p$ and $\varSigma_u$ parameterize the model. Under an MLE approach, these parameters converge to the most likely value of the inputs, effectively discarding distributional information and reducing the computation to point estimates. While this simplification is efficient, it is inherently incompatible with FGL as presented, where the preservation of distributional richness is critical to leveraging temporal variance and uncertainty effectively.

An alternative to the MLE approach is the use of a "surrogate" distribution via variational inference [10]. Variational inference bypasses the computational intractibility of normalization terms (Equation 2) and complex input distributions by introducing a new distribution $q(v)$, which has a standard formcharacterized by its mean and variance.

In the context of FGL, the student and teacher networks can be more accurately defined within a Bayesian framework. Here, the teacher model acts as a well-posed surrogate $T \approx q(v)$, while the student model is treated as a prior, $S = p(v)$. This framing allows the teacher model to serve as a surrogate representation to guide the student model, effectively approximating the posterior $p(v|u)$ with $q(v)$.

The shift from MLE to variational inference involves minimizing the divergence between the surrogate distribution $q(v)$ and the true posterior $p(v|u)$. This divergence is quantified using the Kullback-Leibler (KL) divergence:

$$KL(q(v), p(v|u)) = \int q(v) \ln \frac{q(v)}{p(v|u)} dv \tag{13}$$

While the KL divergence provides a useful metric, it is implausible for a biological network to directly measure this difference due to the same issue of computational intractability of $p(v|u)$ that led to the use of MLE:

$$p(v|u) = \frac{p(u, v)}{p(u)} = \frac{p(u, v)}{\int p(v)p(u|v)dv} \tag{14}$$

Expanding the KL divergence using the above expression, we have:

$$
\begin{aligned}
KL(q(v),p(v|u)) &= \int q(v)\ln\frac{q(v)}{p(v|u)}dv \\
&= \int q(v)\ln\frac{q(v)p(u)}{p(u,v)}dv \\
&= \int q(v)\ln\frac{q(v)}{p(u,v)}dv + \int q(v)\ln p(u)dv
\end{aligned} \tag{15}
$$

Since the surrogate $q(v)$ is a valid probability distribution that integrates to 1, the second term simplifies to $\ln p(u)$, yielding:

$$
KL(q(v),p(v|u)) = \int q(v)\ln\frac{q(v)}{p(u,v)}dv + \ln p(u) \tag{16}
$$

We define the first term as the variational free energy F, which avoids the need for computing the normalization term and as such, the student's prior distribution becomes computationally tractable:

$$
\begin{aligned}
F &= \int q(v)\ln\frac{q(v)}{p(u,v)}dv \\
KL(q(v),p(v|u)) &= F + \ln p(u)
\end{aligned} \tag{17}
$$

since F depends on the surrogate distribution $q(v)$, the parameters which minimize the distance between the surrogate $q(v)$ and the teacher's posterior $p(v|u)$, are identical to those which maximize F, which in turn negates the computation of the normalization term. Substituting F, we obtain:

$$
\ln p(u) = -F + KL(q(v),p(v|u)) \tag{18}
$$

The term $\ln p(u)$, derived above, represents the "surprise" associated with the student's estimate $p(u)$ of the actual value v, and is directly linked to the uncertainty of the student model. Since the KL divergence is strictly non-negative, F acts as a lower bound for the surprise $\ln p(u)$. Consequently, maximizing F corresponds to minimizing surprise or uncertainty of the student $\ln p(u)$, improving the surrogate approximation $q(v)$, and by extension, optimizing the student model with respect to the teacher in the context of FGL.

This concept of surprise is particularly valuable for updating uncertainty parameters within a network performing predictive computations, where $\ln p(u)$ can be written as:

$$
\begin{aligned}
\ln p(u) &= F + KL(q(v),p(v|u)) \\
&= \frac{1}{2}\left[-\ln\Sigma_p - \frac{(\Phi - v_p)^2}{\Sigma_p} - \ln\Sigma_u - \frac{(u - g(\Phi))^2}{\Sigma_u}\right] + C + KL(q(v),p(v|u))
\end{aligned} \tag{19}
$$

Since the KL divergence is strictly non-negative, it can be absorbed into the constant C for simplicity, ensuring the expression focuses on terms directly impacting the uncertainty parameters.

Starting from this definition, we can now derive F to optimize v_p:

$$\begin{aligned}
\frac{\delta F}{\delta v_p} &= \frac{1}{2}\Big(\frac{\delta}{\delta v_p}\big(-\tfrac{(\Phi-v_p)^2}{\Sigma_p}\big) + \frac{\delta}{\delta v_p}\big(-\tfrac{(u-g(\Phi))^2}{\Sigma_u}\big) \\
&\quad + \frac{\delta}{\delta v_p}\big(-\ln\Sigma_u\big) + \frac{\delta}{\delta v_p}\big(-\ln\Sigma_p\big)\Big) + \frac{\delta C}{\delta v_p} \\
&= \frac{1}{2}\,\frac{\delta}{\delta v_p}\big(-\tfrac{(\Phi-v_p)^2}{\Sigma_p}\big) \\
&= \frac{1}{2}\Big(-\tfrac{1}{\Sigma_p}\Big)\frac{\delta}{\delta v_p}(\Phi-v_p)^2 \\
&= -\frac{1}{\Sigma_p}(\Phi-v_p)\frac{\delta}{\delta v_p}(\Phi-v_p) \\
&= -\frac{1}{\Sigma_p}(\Phi-v_p)\,(0-1) \\
&= \frac{\Phi-v_p}{\Sigma_p}\,.
\end{aligned} \tag{20}$$

The same can be done for Σ_p:

$$\begin{aligned}
\frac{\delta F}{\delta \Sigma_p} &= \frac{1}{2}\Big(\frac{\delta}{\delta \Sigma_p}\big(-\tfrac{(\Phi-v_p)^2}{\Sigma_p}\big) + \frac{\delta}{\delta \Sigma_p}\big(-\tfrac{(u-g(\Phi))^2}{\Sigma_u}\big) \\
&\quad + \frac{\delta}{\delta \Sigma_p}\big(-\ln\Sigma_u\big) + \frac{\delta}{\delta \Sigma_p}\big(-\ln\Sigma_p\big)\Big) + \frac{\delta C}{\delta \Sigma_p} \\
&= \frac{1}{2}\Big(-\frac{\delta}{\delta \Sigma_p}\ln\Sigma_p + \big(-(\Phi-v_p)^2\ \tfrac{\delta}{\delta\Sigma_p}(\Sigma_p^{-1})\big)\Big) \\
&= \frac{1}{2}\Big(-\frac{1}{\Sigma_p} + (\Phi-v_p)^2\ \frac{\delta\Sigma_p}{\Sigma_p^2}\Big) \\
&= \frac{1}{2}\Big((\Phi-v_p)^2\ \frac{\delta\Sigma_p}{\Sigma_p^2} - \frac{1}{\Sigma_p}\Big) \\
&= \frac{1}{2}\Big(\tfrac{(\Phi-v_p)^2}{\Sigma_p^2} - \frac{1}{\Sigma_p}\Big).
\end{aligned} \tag{21}$$

Likewise, for Σ_u:

$$\begin{aligned}
\frac{\delta F}{\delta \Sigma_u} &= \frac{1}{2}\Big(\frac{\delta}{\delta \Sigma_u}\big(-\tfrac{(\Phi - v_p)^2}{\Sigma_p}\big) + \frac{\delta}{\delta \Sigma_u}\big(-\tfrac{(u-g(\Phi))^2}{\Sigma_u}\big) \\
&\quad + \frac{\delta}{\delta \Sigma_u}\big(-\ln \Sigma_u\big) + \frac{\delta}{\delta \Sigma_u}\big(-\ln \Sigma_p\big)\Big) + \frac{\delta C}{\delta \Sigma_u} \\
&= \frac{1}{2}\Big(\frac{\delta}{\delta \Sigma_u}\big(-\tfrac{(u-g(\Phi))^2}{\Sigma_u}\big) - \frac{\delta}{\delta \Sigma_u}\ln \Sigma_u\Big) \\
&= \frac{1}{2}\Big(-(u-g(\Phi))^2\,\frac{\delta}{\delta \Sigma_u}(\Sigma_u^{-1}) - \frac{1}{\Sigma_u}\Big) \\
&= \frac{1}{2}\Big(\tfrac{(u-g(\Phi))^2}{\Sigma_u^2} - \frac{1}{\Sigma_u}\Big).
\end{aligned} \tag{22}$$

Yielding the learned parameters of a predictive coding network that performs equivalent computation to FGL as:

$$\begin{aligned}
\frac{\delta F}{\delta v_p} &= \frac{\Phi - v_p}{\Sigma_p} = \epsilon_p \\
\frac{\delta F}{\delta \Sigma_p} &= \frac{1}{2}\left(\frac{(\Phi - v_p)^2}{\Sigma_p^2} - \frac{1}{\Sigma_p}\right) = \frac{1}{2}\left(\epsilon_p^2 - \Sigma_p^{-1}\right) \\
\frac{\delta F}{\delta \Sigma_u} &= \frac{1}{2}\left(\frac{(u-g(\Phi))^2}{\Sigma_u^2} - \frac{1}{\Sigma_u}\right) = \frac{1}{2}\left(\epsilon_u^2 - \Sigma_u^{-1}\right)
\end{aligned} \tag{23}$$

While these equations follow scalar notations, Bogacz [8] showed that they can trivially be extended to vector and matrices forms with neurobiologically plausible implementations that are computationally similar to FGL.

Dataset I/O Summary

**Task**	**Input** x_t	**Target** $y_{t+\ell}$
Seizure pred.	N-ch EEG segment (F Hz)	3-way class (ictal / inter / pre)
Mackey–Glass	1-D chaotic window ($L = 8$)	Scalar at $t + \ell$ (discretised)

References

[1] Beghi, E., Giussani, G., Nichols, E., Abd-Allah, F., Abdela, J., Abdelalim, A., Abraha, H.N., Adib, M.G., Agrawal, S., Alahdab, F., et al.: Global, regional, and national burden of epilepsy, 1990–2016: a systematic analysis for the global burden of disease study 2016. *The Lancet Neurology* **18**(4), 357–375 (2019)

[2] Abbasi, B., Goldenholz, D.M.: Machine learning applications in epilepsy. *Epilepsia* **60**(10), 2037–2047 (2019)

[3] Siddiqui, M.K., Morales-Menendez, R., Huang, X., Hussain, N.: A review of epileptic seizure detection using machine learning classifiers. *Brain informatics* **7**(1), 5 (2020)

[4] Kuhlmann, L., Lehnertz, K., Richardson, M.P., Schelter, B., Zaveri, H.P.: Seizure prediction—ready for a new era. *Nature Reviews Neurology* **14**(10), 618–630 (2018)

[5] Jasper, H.H.: Ten-twenty electrode system of the international federation. *Electroencephalogr Clin Neurophysiol* **10**, 371–375 (1958)

[6] Truong, N.D., Nguyen, A.D., Kuhlmann, L., Bonyadi, M.R., Yang, J., Ippolito, S., Kavehei, O.: Convolutional neural networks for seizure prediction using intracranial and scalp electroencephalogram. *Neural Networks* **105**, 104–111 (2018)

[7] Millidge, B., Seth, A., Buckley, C.L.: Predictive coding: a theoretical and experimental review. arXiv preprint arXiv:2107.12979 (2021)

[8] Bogacz, R.: A tutorial on the free-energy framework for modelling perception and learning. *Journal of mathematical psychology* **76**, 198–211 (2017)

[9] Friston, K.: A theory of cortical responses. *Philosophical transactions of the Royal Society B: Biological sciences* **360**(1456), 815–836 (2005)

[10] Murphy, K.P.: *Machine Learning: a Probabilistic Perspective.* MIT press (2012)
